# Agreement between cardiac cycle time intervals and myocardial velocities measured by automated analysis of color tissue Doppler cine loops and by manual tracing of spectral pulsed‐wave tissue Doppler waveforms of sheep fetal heart under normal and pathological conditions: An experimental study

**DOI:** 10.1111/aogs.70340

**Published:** 2026-08-24

**Authors:** Juulia Lantto, Maria Grazia Morena, Heikki Huhta, Amar Bhide, Jonas Johnson, Juha Räsänen, Ganesh Acharya

**Affiliations:** ^1^ Division of Obstetrics & Gynecology, Department of Clinical Science, Intervention and Technology (CLINTEC) Karolinska Institutet Stockholm Sweden; ^2^ Centre for Fetal Medicine Karolinska University Hospital Stockholm Sweden; ^3^ Translational Research Group Oulu University Hospital and Oulu University Oulu Finland; ^4^ St. George's Hospital and City‐St. George's University of London London UK; ^5^ Helsinki University Hospital and University of Helsinki Helsinki Finland

**Keywords:** aortic constriction, cardiac time intervals, color tissue Doppler, fetal hypoxemia, fetal sheep model, myocardial velocities, spectral pulsed‐wave tissue Doppler, tissue Doppler imaging

## Abstract

**Introduction:**

This study evaluated agreement between automated analysis of color tissue Doppler (cTDI) cine loops and manual spectral pulsed‐wave tissue Doppler (pwTDI) measurements of fetal cardiac cycle time intervals and myocardial velocities under normal and pathological conditions in an experimental model using fetal sheep.

**Material and Methods:**

In 10 sheep fetuses, 25%–50% partial ascending aorta constriction was performed, and in nine, utero‐placental blood flow was reduced by maternal uterine artery occlusion. Four fetuses served as controls. Experiments were performed after 4 to 5 days recovery, and cTDI cine loops and pwTDI waveforms were sequentially obtained during baseline and during fetal hypoxemia induced by maternal hypo‐oxygenation. cTDI cine loops were processed using an automated, ECG‐independent algorithm, whereas pwTDI waveforms were manually traced to measure cardiac cycle time intervals and myocardial velocities.

**Results:**

Fetal heart rate (FHR) showed excellent agreement between methods (intraclass correlation coefficient, ICC = 0.94). In contrast, myocardial velocities and cardiac time intervals demonstrated poor‐to‐moderate absolute agreement (ICC range 0.20–0.63). Bland–Altman analysis revealed systematic modality‐specific offsets: the automated method yielded consistently lower velocities and longer isovolumic intervals. These offsets were stable across physiological states, with no metric showing phase‐dependent changes in bias after Benjamini–Hochberg false discovery rate (FDR) correction (all *q* > 0.05). Despite limited absolute agreement, consistency and correlation (ICC(C,1) and Pearson r) were markedly higher than absolute agreement, indicating strong preservation of relative ranking across methods. Lin's concordance correlation coefficient (CCC) showed a similar pattern, with high concordance for FHR (CCC = 0.936) and lower concordance for most other metrics.

**Conclusions:**

Automated cTDI is not directly interchangeable with manual pwTDI for absolute measurements of sheep fetal myocardial velocities or time intervals due to persistent modality‐specific offsets. However, the automated method provides reproducible relative tracking and stable performance across different physiological conditions. Automated cTDI is therefore well suited for longitudinal assessments when interpreted using method‐specific baselines or calibration. FHR was the only parameter demonstrating near‐interchangeable performance.

## Graphical abstract

Automated cTDI in sheep fetuses provided reproducible measurements of myocardial velocities and heart rate that align well with manual pulsed‐wave TDI. For cardiac cycle duration metrics, the two modalities are not interchangeable. These findings support that automated cTDI is well suited for longitudinal assessment under both physiological and pathological situations when interpreted using method‐specific baselines or calibration.

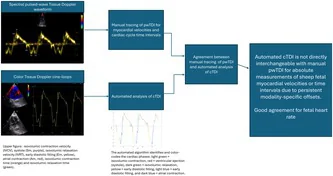

**Abbreviations**
Am: atrial contraction
AmDur: atrial‐contraction duration
AoC: ascending aorta constriction
CCC: concordance correlation coefficient
CI: confidence interval
cTDI: color tissue Doppler imaging
Em: early diastolic filling
EmDur: early diastolic filling duration
FHR: fetal heart rate
FTDur: filling‐time duration
ICC: intraclass correlation coefficient
IVCT: isovolumic contraction time
IVCV: isovolumic contraction velocity
IVRT: isovolumic relaxation time
IVRV: isovolumic relaxation velocity
LoA: limits of agreement
pwTDI: spectral pulsed wave Doppler imaging
ROI: region of interest
SD: standard deviation
Sm: ventricular ejection
SmDur: ventricular ejection duration
UtA: uterine artery

**Key message**

Automated color Tissue Doppler on fetal sheep provided reproducible measurements of myocardial velocities that align well with pulsed‐wave Tissue Doppler in terms of relative rankings across normal and pathophysiological states. However, absolute values are not interchangeable due to modality‐specific differences. Automated cTDI could be used for longitudinal assessments of fetal sheep cardiac function.

## 1 INTRODUCTION

Tissue Doppler imaging (TDI), using both spectral pulsed‐wave (pwTDI) and color (cTDI) modalities, is a key tool for the quantitative assessment of fetal cardiac function.^1^, ^2^ Both techniques are feasible in human fetuses, and gestational age‐specific reference values have been established.^3^, ^4^, ^5^, ^6^, ^7^ TDI has proven invaluable for detecting early signs of cardiac dysfunction across a wide range of clinical and experimental settings. For instance, pwTDI has been shown to identify compromised myocardial function in conditions, such as fetal growth restriction,^8^, ^9^, ^10^ congenital heart defects,^11^, ^12^ and in fetuses of diabetic mothers.^13^, ^14^ Similarly, cTDI has shown potential in distinguishing healthy from compromised human fetuses in twin‐twin transfusion syndrome^15^ and in experimental animal models of fetal hypoxemia and altered cardiac load.^16^, ^17^, ^18^, ^19^, ^20^

One of the advantages of using cTDI is that the myocardial velocity information can be obtained from the entire sector of the cardiac image, and multiple regions of interest (ROIs) can be examined simultaneously.^21^ Although offline analysis of the cTDI images to get parameters of myocardial velocities and cardiac cycle time intervals from individual segments is time‐consuming and highly operator dependent, this could be overcome by automated analysis of cTDI cine loop images of the fetal heart which also allows measurement and averaging of parameters obtained from several cardiac cycles. We have previously shown the feasibility of the automated analysis of cTDI derived left ventricular (LV), septal and right ventricular (RV) cTDI myocardial velocity traces in human fetuses at different gestational ages and under altered loading conditions following intrauterine blood transfusion.^15^, ^22^, ^23^, ^24^ However, whether cTDI and pwTDI derived parameters of fetal cardiac function are similar and/or correlate with each other is not known.

The aim of our current study was to evaluate the agreement and correlation between the measurements of fetal myocardial velocities and mechanical cardiac cycle time intervals measured by spectral pwTDI waveform analysis performed manually by a human observer (current gold standard method) and those measured using an automated, rule‐based algorithm to analyze tissue velocities from the cTDI cine loops of the four‐chamber view of the heart under different pathophysiological conditions in a near‐term experimental sheep model. We hypothesized that the automated analysis of the cTDI images is comparable to the standard analysis method of manual tracing of pwTDI velocity waveforms by human observer.

## 2 MATERIAL AND METHODS

A total of 23 pregnant sheep of Aland landrace sheep (Lammastila Sikka Talu, University of Turku, Rymättylä, Finland) with time‐dated pregnancies were included in this study. Sheep were transported 2 weeks before the experimentation to the Oulu Laboratory Animal Centre at the University of Oulu, Finland, to allow for adaptation. Sheep were monitored several times daily by a veterinarian, animal technicians and investigators for signs of pain, distress, injury, or disease. The focus was to ensure the well‐being of animals and to minimize pain.

### 2.1 Ethics approval

The study protocol was approved by the National Animal Experiment Board of Finland (ESAVI/1007/04.10.07/2014 on date March 13, 2014, and ESAVI/7840/04.10.07/2017 on date September 7, 2017). The animal care and experimental procedures were conducted according to the national legislation,^25^, ^26^ the European Union Directive 2010/63/EU^27^ and according to ARRIVE 2.0 guidelines.^28^

### 2.2 Fetal instrumentation

Sheep fetuses were instrumented at gestational age 118–130 (term 145 days). The ewes were premedicated with intramuscular ketamine (Ketaminol vet; Intervet, Boxmeer, The Netherlands) 2 mg/kg and midazolam (Midazolam Hameln; Hameln Pharmaceuticals, Hameln, Germany) 0.2 mg/kg. Maternal jugular vein was cannulated to obtain intravenous (i.v.) access and an infusion of lactated Ringer's solution was given at a rate of 200 mL/h. The ewe was given a single dose of prophylactic antibiotic, ampicillin 1 g i.v. (A‐Pen Orion, Orion Pharma Oyj, Espoo, Finland). General anesthesia was induced with i.v. propofol (Profast, Baxter Holding B.V, Utrecht, Netherlands) 4–7 mg/kg, and the ewe was intubated. The anesthesia was maintained with 2%–2.5% sevoflurane (AbbVie S.r.l., Campoverde di Aprilia, Italy) in oxygen‐air mixture and i.v. propofol‐infusion at a rate of 0.5–1 mL/h (Profast, Baxter Holding B.V, Utrecht, Netherlands). Maternal auricular artery was cannulated with a 22F cannula for invasive blood pressure monitoring. Analgesia was provided with 50 μg fentanyl i.v. (Fentanyl Hameln, Hameln pharma gmbh, Hameln, Germany) prior to and every 1 h during the surgery or earlier if required, based on maternal blood pressure and heart rate changes during surgical stimuli. In case of a twin pregnancy, only one fetus was instrumented.

Fourteen fetuses were included in the ascending aorta constriction (AoC) group. Fetuses were randomly assigned to different study groups: controls (*n* = 4), 25% reduction (*n* = 3) or 50% reduction (*n* = 7) in ascending aortic area. Sheep was placed supine with a right lateral tilt. Fetal ascending aorta was identified using a Vivid 7 Dimension ultrasound system (GE Vingmed Ultrasound, Horten, Norway) with a 10 MHz‐phased‐array transducer and the inner diameter of the ascending aorta (D) was measured. A lower midline incision was performed to access the uterus and the fetus through hysterotomy. A left lateral thoracotomy was then performed on the fetus, and the pericardial sac was opened to expose the great arteries. Ascending aorta was identified, and a silicone band was placed around the ascending aorta. For 25% reduction in ascending aorta area, the length of the silicone band was calculated as $C=\left(\frac{D\sqrt{3}}{2}\right)\times \prod$, and for 50% reduction in aortic area, the length of the silicone band was calculated as $C=\left(\frac{D}{\sqrt{2}}\right)\times \prod$. No silicone band was placed around the ascending aorta in the control animals. The pericardium and thoracotomy were closed with sutures. Thereafter, a nonocclusive polyvinyl catheter was inserted into the left axillary artery and vein, respectively. A three‐lead 28‐gauge silver‐coated copper ECG wire (New England Wire Tech., Lisbon, NH) was placed subcutaneously on the fetal chest.

Nine fetuses were included in the uterine artery (UtA) occlusion group. Sheep were placed supine with a lateral tilt, and a lower midline incision was performed. First, an inflatable occluder was placed around the maternal left UtA and a 6‐mm transit‐time ultrasonic flow probe (Transonic Systems; Ithaca, NY) was placed distally from the occluder around the left UtA to confirm that there was no blood flow through the UtA when the occluder was later inflated on the experimental day. Thereafter, the fetal lower body was exposed through the uterotomy and a nonocclusive polyvinyl catheter was placed in the femoral artery and vein, respectively.

A separate polyvinyl catheter was placed in the amniotic cavity to measure amniotic fluid pressure. Lost amniotic fluid was replaced with warm sterile 0.9% saline. Injection of penicillin G (1 million units; Geepenil; Orion Oyj, Espoo, Finland) was administered to the fetus. The uterus was closed with a purse string‐suture. After the closure of the laparotomy incision, the catheters and cables were tunneled subcutaneously and exteriorized through a small incision in the ewe's flank. Postoperative analgesia was provided with local anesthesia of bupivacaine (Bupivacaine Accord 5 mg/mL; Accord Healthcare B.V., Utrecht, The Netherlands) injected locally into the surgical wounds and 20 μ/h buprenorphine transdermal patch (Buprenorphine depot patch, 20 μg/h, ratiopharm GmbH, Ulm, Germany) that was placed in ewe's antebrachium for a minimum of 24 h before the start of surgery.

### 2.3 Experimental protocol

After a 5‐day recovery period at 122–135 (mean (SD) 127 (4)) gestational days, general anesthesia was induced with a single bolus of propofol and maintained with sevoflurane only. The sevoflurane concentration was titrated to keep the ewe's heart rate and blood pressure normal and allow for ultrasound imaging without discomfort while minimizing the physiological alterations associated with its use. Before induction, each ewe was prehydrated with 1 liter of lactated Ringer solution, followed by a fixed infusion of lactated Ringer solution at a rate of 200 mL/h throughout the experiment. A 16‐gauge polyurethane catheter was inserted into the maternal femoral artery to measure maternal arterial blood pressure and to obtain arterial blood gas samples. The ewe was placed supine with a lateral tilt and allowed to stabilize for 30 min before the baseline measurements were taken.

In the AoC group, after the baseline measurements were taken, maternal hypo‐oxygenation was induced by reducing oxygen concentration in a rebreathing circuit to reach a maternal arterial oxygen saturation level of 70%–80%. This was confirmed by an arterial blood gas sample. Data acquisition of invasive measurements (blood pressure and fetal blood gases) and fetal echocardiography was performed at baseline, 30 min (Timepoint 1), 60 min (Timepoint 2) and 120 min (Timepoint 3) after confirmed maternal hypo‐oxygenation. Thereafter, maternal oxygenation was restored and after 30 min of maternal normoxemia, data was collected (Timepoint 4).

In the UtA occlusion group, after the baseline measurements were taken, the UtA occluder was filled with sterile water. Total occlusion of the UtA was confirmed with zero flow assessed with the transit‐time ultrasonic flow probe. Data acquisition of invasive measurements (maternal and fetal blood pressure and arterial blood gas values) and fetal echocardiography was performed at baseline and at 60 min (Timepoint 1), 90 min (Timepoint 2), 120 min (Timepoint 3), 150 min (Timepoint 4), and 180 min (Timepoint 5) after the Ut A occluder had been inflated. In five fetuses, additional maternal hypo‐oxygenation (to maternal saturation level of 70%–80%) was induced after 60 min of UtA occlusion and in four fetuses after 120 min of UtA occlusion.

### 2.4 Invasive data

Arterial blood gas values were corrected for 39°C. Amniotic pressure was subtracted from the recorded fetal invasive blood pressure.

### 2.5 Ultrasonographic data acquisition

Fetal echocardiography was performed using a Vivid 7 Dimension ultrasound system (GE Vingmed Ultrasound AS, Horten, Norway) with a 7 MHz‐phased‐array transducer. The high‐pass filter was set at minimum. All examinations were performed by an experienced ultrasonographer (J.R.). An apical or basal four‐chamber view of the fetal heart was obtained and a 6‐s cine loops of cTDI images were stored digitally for offline measurement using automated algorithm (Method A). Approximately 30 s later, myocardial velocity waveforms were obtained using spectral pwTDI (Method M) by placing the Doppler sample volume (size 1‐2 mm) at the ventricular wall at the level of atrioventricular annulus and cine loops of at least 5–10 consecutive cardiac cycles were recorded. The Doppler insonation angle was kept as close to zero as possible (always <15° with respect to the long axis of the heart). The sector width was adjusted to obtain as high frame rate as possible. Similar dataset was obtained at each phase during the experiment.

At the end of the experiment, the fetus and the ewe were euthanized with an intravenous overdose (100 mg/kg) of pentobarbital sodium (Mebunat vet; Orion Oyj, Espoo, Finland), and fetal weight and sex were determined.

### 2.6 Offline analysis of the ultrasonographic data

Spectral pwTDI and cTDI recordings were analyzed offline by a single investigator (JL) using EchoPAC version 201 (GE Vingmed Ultrasound AS, Horten, Norway).

### 2.7 Manual analysis of pwTDI waveforms (method M)

**FIGURE 1:**
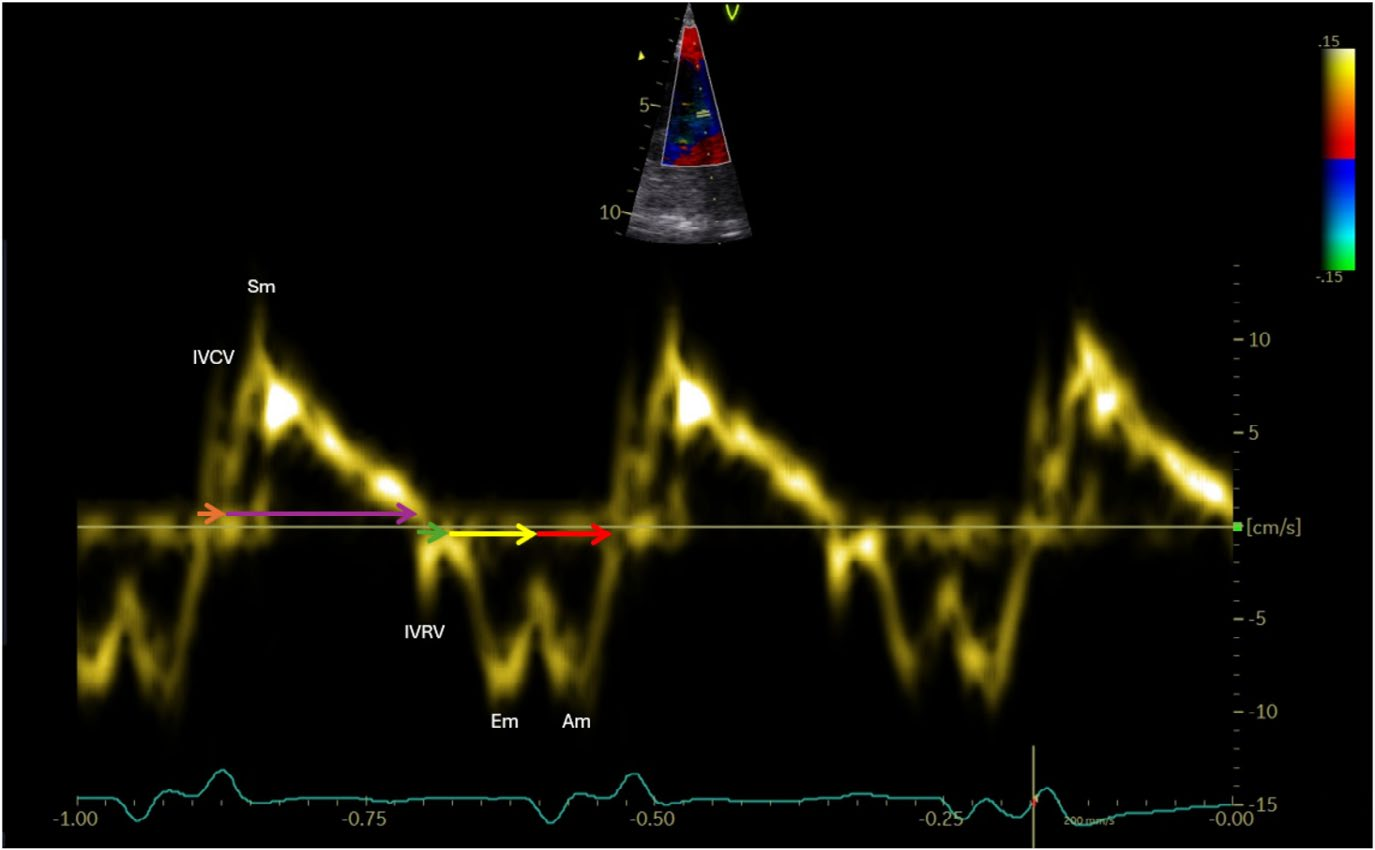
Example of spectral pulsed‐wave Doppler (pwTDI) waveform of fetal sheep right ventricle at atrioventricular valve level showing isovolumic contraction velocity (IVCV), systole (Sm, purple), isovolumic relaxation velocity (IVRT), early diastolic filling (Em, yellow), atrial contraction (Am, red), isovolumic contraction time (orange) and isovolumic relaxation time (green). Fetal heart rate 173 bpm, sweep speed 200 mm/s.

For each recording, a representative pwTDI trace was selected for analysis. Cardiac cycle phases were identified to measure peak myocardial velocities during atrial‐contraction (Am), ventricular ejection (Sm), and early diastolic filling (Em). Time‐interval metrics were obtained, including isovolumic contraction time (IVCT), isovolumic relaxation time (IVRT), early diastolic filling duration (EmDur), atrial‐contraction duration (AmDur), and filling‐time duration (FTDur) defined as the sum of EmDur and AmDur. Fetal heart rate (FHR) was also recorded (Figure 1). Measurements were performed on three consecutive cardiac cycles, and the mean value was used for analysis.

### 2.8 Automated analysis of cTDI cine loops (Method A)

**FIGURE 2:**
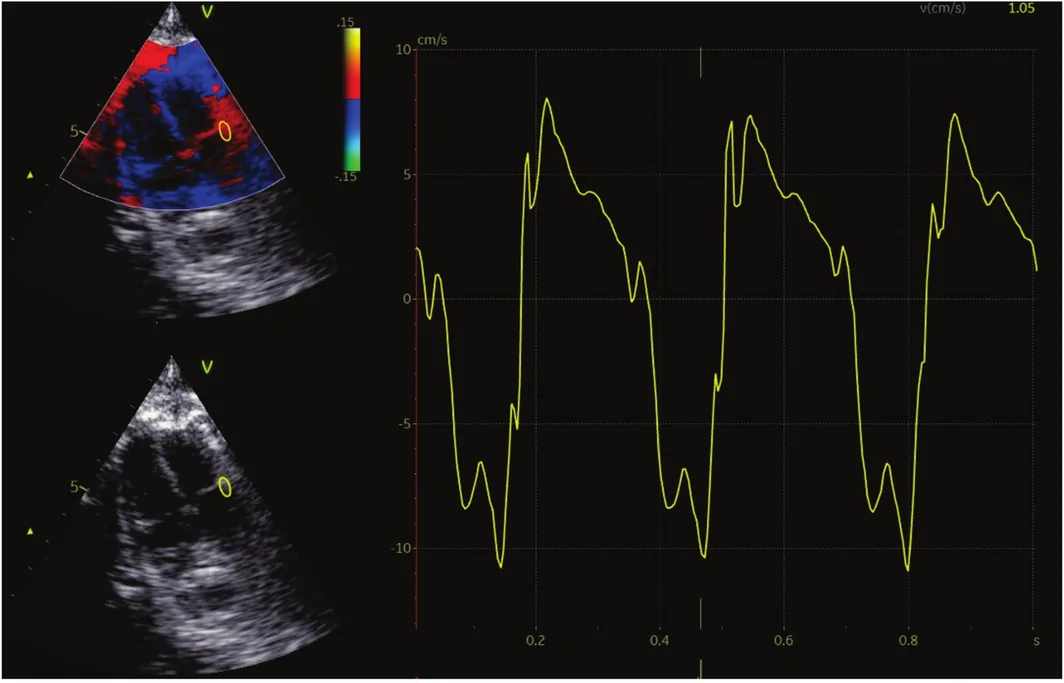
Placement of the region of interest (6 × 3 mm) on the fetal right ventricular wall at the atrioventricular valve plane level to measure the color tissue Doppler imaging (cTDI) waveform with EchoPAC. This waveform was then exported and transferred to MATLAB for analysis by automated algorithm (Figure 3).

A region of interest (ROI; 6 × 3 mm) was placed at the atrioventricular plane in the ventricular wall (Figure 2), consistent with the methodology described previously by Herling et al.^22^ A three‐sample moving‐average smoothing filter was applied in EchoPAC. Myocardial velocity traces were exported as text files containing full raw velocity data and processed in MATLAB (R2023a, MathWorks, Natick, MA, USA) using a custom, fully automated rule‐based tissue velocity imaging (TVI) algorithm. No manual annotation is required once traces are imported. In AoC group, the left ventricular cTDI cine loops were affected by the instrumentation (constriction of the ascending aorta) and caused severe technical interference. Therefore, only RV cTDI cine loops and pwTDI waveforms were used for the analysis. In the UtA Occlusion group, both LV and LV cTDI cine loops and spectral pwTDI waveforms were analyzed.

**FIGURE 3:**
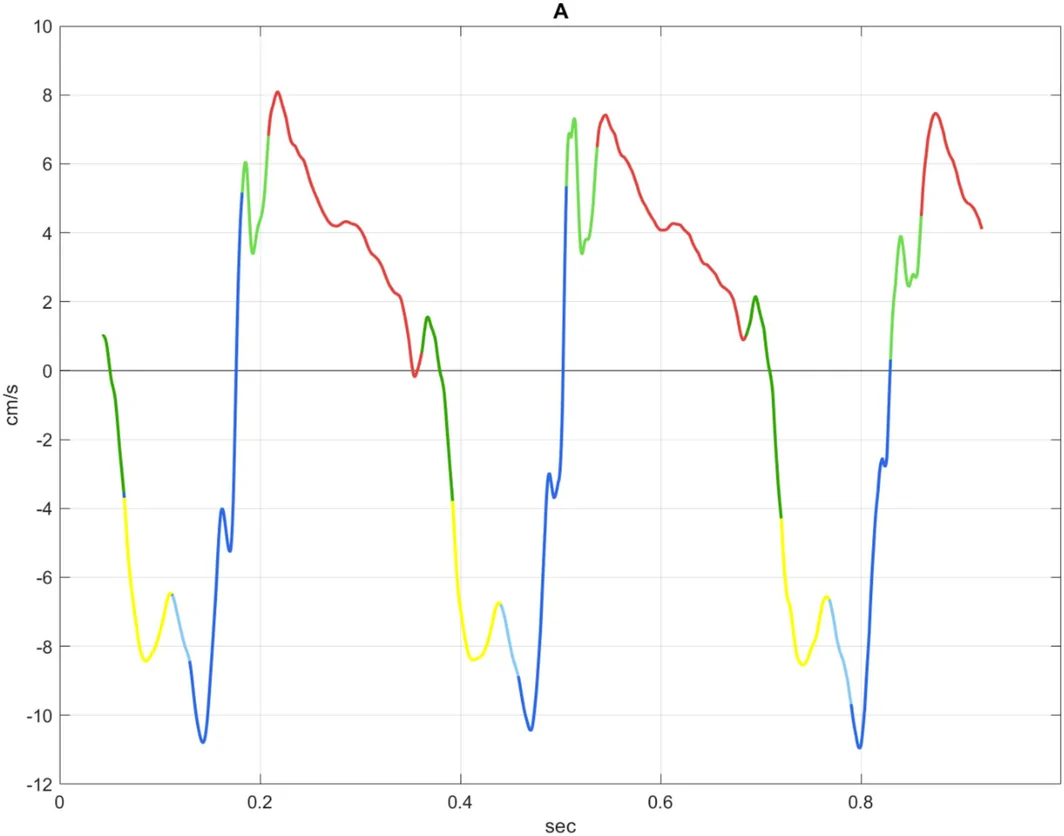
Automated segmentation of a color tissue Doppler imaging (cTDI) waveform from the fetal right ventricle. The plot shows myocardial velocity (cm/s) versus time (s). The waveform displays a pattern typical for the fetal right ventricle at high heart rates. The automated algorithm identifies and color‐codes the cardiac phases: Light green = isovolumic contraction (pre‐ejection), red = ventricular ejection (systole), dark green = isovolumic relaxation (post‐ejection), yellow = early diastolic filling (rapid component), light blue = early diastolic filling (fast component), and dark blue = atrial contraction. In this apical view, positive velocities represent annular motion toward the apex.

The algorithm analyzes each beat individually while leveraging inter‐beat consistency to refine event identification. Phase boundaries are detected from changes in myocardial velocity and its derivatives (acceleration and jerk), incorporating zero‐crossings and waveform morphology. This signal‐processing framework—aligned with the dynamic atrioventricular‐plane displacement model—enables robust, ECG‐independent detection of cardiac phases. The algorithm delineates atrial contraction, isovolumic contraction (pre‐ejection), ventricular ejection (systole), isovolumic relaxation (post‐ejection), and early diastolic filling (rapid and slow components) (Figure 3). The onset of atrial contraction, representing the initial displacement of the atrioventricular plane, defines the start of the cardiac cycle.

### 2.9 Statistical analyses

#### 2.9.1 Data presentation and pairing

Continuous physiological variables (e.g., fetal weight, maternal arterial pressure, and arterial blood gas values) are reported as mean (SD). Variables with non‐normal distributions (fetal mean arterial pressure [MAP] and fetal arterial blood gas values) are summarized as median (range).

Ultrasonographic outcomes were paired within each observation block, comparing the automated algorithm (Method A) with manual pwTDI analysis (Method M). When multiple beats were available, values were aggregated to the median per method before pairing. The paired observation blocks were obtained from 22 fetuses (1–9 blocks per animal); to account for this clustered structure, a sensitivity analysis was performed in which intraclass correlation coefficient (ICC) 95% confidence intervals (CIs) were recomputed using an animal‐clustered bootstrap (resampling fetuses rather than individual observations) and the ICC and Lin's concordance correlation coefficient (CCC) were re‐estimated after aggregating each fetus to its median paired value. Experimental phases were encoded as a categorical variable: 0 = RV baseline, 1 = RV hypoxemia, 2 = RV aortic constriction, 3 = LV baseline, 4 = LV hypoxemia. In the AoC group, the LV cTDI cine loops were affected by the instrumentation and therefore could not be included in the analysis. Agreement metrics (ICC, CCC and Bland–Altman) were computed from all available paired observations across all phases, whereas the phase‐based comparisons performed to examine the effects of fetal oxygenation status on model performance were restricted to phases {0, 1, 3, 4}.

#### 2.9.2 Agreement and reproducibility

Intermethod reliability was quantified using ICC derived from a two‐way mixed‐effects model (single measurement), reporting ICC(A,1) for absolute agreement and ICC(C,1) for consistency. A mixed‐effects model was used because the two raters represent fixed, pre‐defined measurement methods rather than a random sample of raters; absolute agreement ICC(A,1) was the primary index because the question of interest was the interchangeability of absolute values, with consistency ICC(C,1) reported to separate systematic offsets from rank/scaling agreement.

As a complementary measure of agreement, Lin's CCC was computed, including its components of precision (Pearson's r) and accuracy (bias‐correction factor C_b). For both ICC and CCC, 95% CIs were obtained using percentile bootstrapping (1000 resamples). Agreement was interpreted using standard thresholds: <0.50 poor, 0.50–0.74 moderate, 0.75–0.89 good, and ≥0.90 excellent.

#### 2.9.3 Method bias and Bland–Altman analysis

Absolute agreement was assessed using Bland–Altman statistics,^29^ defining bias as the mean difference (Algorithm—Manual) and computing limits of agreement (LoA = bias ±1.96 SD). Proportional bias was evaluated by regressing the between‐method difference on the mean using ordinary least squares (OLS); the regression slope was tested against zero and reported with its 95% CI. For descriptive assessment of the method relationship, robust Theil–Sen regression was additionally performed with algorithm‐derived values regressed on manual values; the resulting slopes are reported in the Results.

#### 2.9.4 Phase effects and baseline offsets

For RV baseline (Phase 0) and LV baseline (Phase 3), bias was tested against zero using one‐sample *t*‐tests. To assess whether bias differed across physiological states, a one‐way ANOVA was performed for each metric with bias as the dependent variable and phase as the independent factor (restricted to Phases 0, 1, 3, and 4). Effect sizes were reported as *η* ^2^. Pre‐specified contrasts compared RV baseline (0) vs. RV hypoxemia (1) and LV baseline (3) vs. LV hypoxemia (4), estimated using OLS with HC3 robust standard errors.

#### 2.9.5 Temporal synchronization analysis

A time‐lag variable (TimeGapSec) was constructed from block‐level timestamps (manual vs. automated). The association between time lag and bias was evaluated using Spearman's rank correlation and a multivariable OLS model controlling for phase (HC3 robust SE).

#### 2.9.6 Multiple testing and software

Where multiple testing was performed across metrics, *p*‐values were adjusted using the Benjamini–Hochberg false discovery rate procedure, with *q* < 0.05 considered significant. Significant global ANOVAs were followed by Tukey's HSD post hoc tests where applicable. Analyses were performed in Python 3.11 using Pingouin (ICC, ANOVA, Tukey), Statsmodels (regression with HC3 SE), SciPy (core statistics), and scikit‐learn (Theil–Sen regression).

## 3 RESULTS

The mean (SD) fetal weight was 2710 (481) g and ranged from 1910 to 3665 g. Fourteen fetuses were female (mean weight 2544 (385) g) and nine were male (mean weight 2968 (501) g). Fetal arterial blood gas values are presented in Table 1. In the AoC group, median MAP in the axillary artery at baseline was 31 mmHg ranging from 12 mmHg in the fetuses with 50% constriction to 58 mmHg in control fetuses. At the last hypoxemia phase (Timepoint 3), the median MAP was 34 (range 11–48), respectively. In the UtA occlusion group, median (range) MAP in the descending aorta was 38 (30–43) mmHg at baseline, and during the last hypoxemia phase (Timepoint 5) MAP was 46 (30–62) mmHg.

**TABLE 1:** Fetal arterial blood gas values during baseline and during the experiment. Values presented as median (range).

	Study group	Baseline	Timepoint 1	Timepoint 2	Timepoint 3	Timepoint 4	Timepoint 5
pH	AoC	7.28 (7.19–7.37)	7.23 (7.09–7.36)	7.17 (6.96–7.37)	7.16 (6.77–7.36)	7.15 (6.75–7.39)	
	Control	7.27 (7.20–7.30)	7.26 (7.24–7.29)	7.27 (7.20–7.29)	7.25 (7.12–7.29)	7.26 (7.11–7.27)	
	UtA Occl	7.33 (7.22–7.44)	7.31 (7.22–7.35)	7.30 (7.17–7.35)	7.26 (7.06–7.34)	7.27 (7.04–7.33)	7.15 (6.93–7.27)
pCO_2_ (kPa)	AoC	8.24 (6.66–9.68)	9.36 (5.46–10.73)	9.30 (5.54–13.85)	9.27 (5.53–17.33)	8.36 (6.10–15.36)	
	Control	6.98 (6.93–9.05)	7.10 (6.47–7.72)	6.77 (5.35–7.74)	6.73 (5.50–7.59)	6.90 (5.39–8.24)	
	UtA Occl	7.00 (6.31–7.92)	7.55 (7.01–9.60)	7.33 (6.55–9.22)	7.85 (5.40–9.24)	7.42 (6.50–8.35)	7.09 (6.26–8.52)
pO_2_ (kPa)	AoC	2.4 (12.2–3.4)	1.3 (1.1–1.8)	1.3 (0.9–1.6)	1.5 (1.0–3.0)	2.1 (1.2–3.3)	
	Control	3.0 (1.9–4.0)	1.4 (1.2–1.6)	1.7 (1.2–1.8)	1.1 (0.9–1.3)	3.0 (2.3–3.8)	
	UtA Occl	3.1 (2.1–5.1)	2.65 (1.3–3.7)	1.8 (0.9–3.2)	1.7 (1.0–2.8)	1.5 (0.9–2.1)	1.5 (1.2–2.3)
Base excess (mmol/L)	AoC	1 (−7 – 8)	−4 (−9 – 3)	−9 (−9 – −1)	−12 (−16–1)	−10 (−20 – −1)	
	Control	−2 (−3 – −1)	−3^a^	−7 (−7 – −4)	−7 (−11 – −5)	−7 (−10 – −4)	
	UtA Occl	2 (−4 – 4)	3 (−3 – 4)	2 (−7 – 4)	−3 (−14 – 4)	−6 (−16 – 0)	−10 (−20 – −4)
Lactate (mmol/L)	AoC	2.07 (1.10–11.1)	5.11 (2.54–15.9)	5.53 (4.8–17.8)	12.61 (10.68–19.5)	11.97 (8.33–15.24)	
	Control	1.06 (0.97–1.33)	2.81 (2.05–3.57)	5.11 (2.96–6.65)	6.73 (4.27–9.29)	4.95 (3.44–9.52)	
	UtA Occl	1.54 (0.98–3.61)	1.90 (1.35–7.02)	2.80 (1.59–8.46)	6.29 (1.8–11.56)	8.59 (4.9–13.71)	12.00 (8.3–16.53)

*Note*: *AoC*, ascending aorta constriction group, Timepoint 1 = maternal hypo‐oxygenation 30 min, Timepoint 2 = maternal hypo‐oxygenation 60 min, Timepoint 3 = maternal hypo‐oxygenation 120 min, Timepoint 4 = maternal normoxemia. *Control* group, Timepoint 1 = maternal hypo‐oxygenation 30 min, Timepoint 2 = maternal hypo‐oxygenation 60 min, Timepoint 3 = maternal hypo‐oxygenation 120 min, Timepoint 4 = maternal normoxemia. *UtA Occl*, uterine artery occlusion group, Timepoint 1 = UtA occlusion 60 min, Timepoint 2 = UtA occlusion 90 min, Timepoint 3 = UtA occlusion 120 min, Timepoint 4 = UtA occlusion 150 min, Timepoint 5 = UtA occlusion 180 min.

Abbreviations: kPa, kilo Pascal; mmHg, millimeter of Mercury; mmol/L, millimoles per liter; pCO_2_, partial pressure of carbon dioxide; pO_2_, partial pressure of oxygen.

^a^ All measured values same.

Ten out of fourteen fetuses survived the whole experiment until the recovery period in the AoC group (two fetuses died during hypoxemia 120 min phase and two died during the recovery phase). All nine fetuses in the UtA occlusion group reached the final phase.

Maternal arterial blood gas values are presented in Table 2. Maternal heart rate was mean (SD) 113 (15), systolic blood pressure was 112 (15), diastolic blood pressure was 75 (13), and MAP was 88 (16) at baseline, and these parameters were stable throughout the experiment.

**TABLE 2:** Maternal arterial blood gas values at baseline and during the experiment. Values presented as mean (standard deviation).

	Study group	Baseline	Timepoint 1	Timepoint 2	Timepoint 3	Timepoint 4	Timepoint 5
pH	AoC	7.38 (0.05)	7.41 (0.04)	7.40 (0.05)	7.40 (0.06)	7.35 (0.07)	
	UtA Occl	7.35 (0.04)	7.33 (0.05)	7.35 (0.05)	7.34 (0.04)	7.33 (0.06)	7.34 (0.06)
pCO_2_ (kPa)	AoC	5.42 (0.32)	4.80 (0.44)	4.77 (0.45)	4.56 (0.34)	5.14 (0.44)	
	UtA Occl	5.47 (0.56)	5.60 (0.53)	4.94 (0.37)	5.12 (0.32)	4.94 (0.32)	4.90 (0.41)
pO_2_ (kPa)	AoC	26.38 (8.21)	6.54 (2.12)	6.28 (1.07)	6.28 (1.77)	21.95 (6.74)	
	UtA Occl	19.64 (7.85)	15.36 (5.64)	5.80 (1.76)	9.10 (4.96)	4.55 (0.79)	4.86 (0.41)
Base excess (mmol/L)	AoC	−1 (3)	−2 (3)	−3 (3)	−4 (4)	−4 (3)	
	UtA Occl	−3 (3)	−4 (2)	−5 (2)	−5 (2)	−7 (3)	−6 (2)

*Note*: *AoC*, ascending aorta constriction group, Timepoint 1 = maternal hypo‐oxygenation 30 min, Timepoint 2 = maternal hypo‐oxygenation 60 min, Timepoint 3 = maternal hypo‐oxygenation 120 min, Timepoint 4 = maternal normoxemia. *UtA Occl*, uterine artery occlusion group, Timepoint 1 = UtA occlusion 60 min, Timepoint 2 = UtA occlusion 90 min, Timepoint 3 = UtA occlusion 120 min, Timepoint 4 = UtA occlusion 150 min, Timepoint 5 = UtA occlusion 180 min.

Abbreviations: bpm, beats per minute; kPa, kilo Pascal; mmHg, millimeter of Mercury; mmol/L, millimoles per liter; pCO_2_, partial pressure of carbon dioxide; pO_2_, partial pressure of oxygen.

A total of 95 observation blocks were acquired and used for the agreement analyses. Two cTDI recordings required minor adjustment due to poor initial signal quality, but no datasets were excluded. Because signal quality varied between metrics, the number of valid paired measurements available for each parameter ranged from 62 to 95. For the automated cTDI analysis, a mean of 8.2 ± 2.9 beats per cine loop was available for processing. For manual pwTDI analysis, three consecutive cardiac cycles were measured and averaged for each recording.

### 3.1 Intermethod agreement (ICC analysis)

**FIGURE 4:**
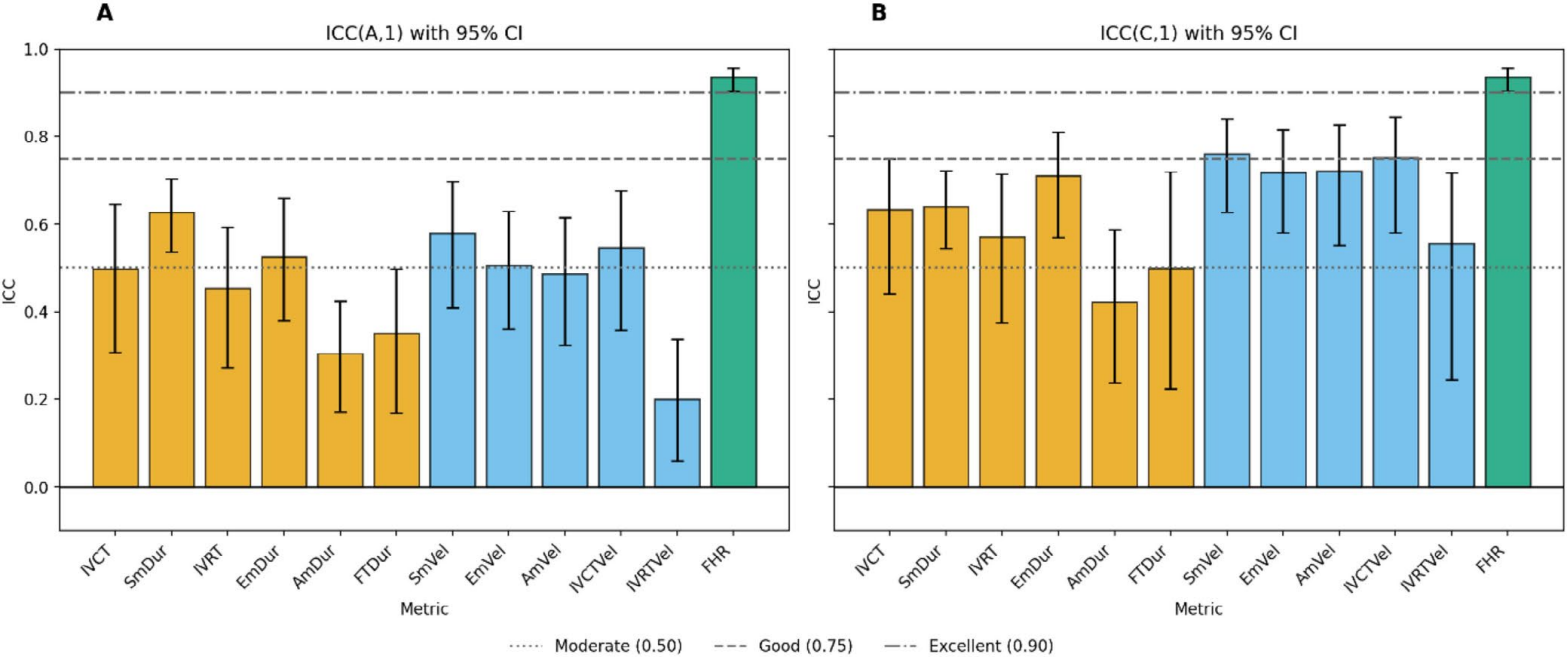
Intraclass correlation coefficients (ICCs) with 95% confidence intervals (CIs) for all metrics. Panel A shows ICC(A,1) (absolute agreement) and Panel B shows ICC(C,1) (consistency). Horizontal dashed reference lines mark conventional thresholds: 0.50 (moderate, orange), 0.75 (good, light blue), and 0.90 (excellent, green). Bars are colored by metric class: Durations (orange), velocities (blue), and FHR (green). AmDur, atrial‐contraction duration; AmVel, atrial‐contraction velocity; EmDur, early diastolic filling duration; EmVel, early diastolic filling velocity; FHR, fetal heart rate; FTDur, filling‐time duration; IVCT, isovolumic contraction time; IVRT, isovolumic relaxation time; SmDur, systolic (ejection) duration; SmVel, systolic (ejection) velocity; IVCTVel/IVRTVel, velocities during isovolumic contraction/relaxation.

The ICC was calculated to evaluate agreement between the human observer (M) and the algorithm (A) across 12 metrics (Table 3, Figure 4). Fetal heart rate showed excellent agreement (ICC = 0.94; 95% CI 0.90–0.96). For time intervals, agreement varied. Systolic duration (SmDur) showed moderate agreement (ICC(A,1) = 0.63; 95% CI 0.54–0.70), as did isovolumic contraction time (IVCT, ICC = 0.50) and early diastolic duration (EmDur, ICC = 0.53). However, isovolumic relaxation time (IVRT), atrial‐contraction duration (AmDur), and total filling time (FTDur) demonstrated poor agreement, with ICC values of 0.45, 0.30, and 0.35, respectively. Tissue velocities showed moderate agreement for peak systolic velocity (SmVel, ICC = 0.58), early diastolic velocity (EmVel, ICC = 0.51), and isovolumic contraction velocity (IVCTVel, ICC = 0.55). Atrial‐contraction velocity (AmVel) and isovolumic relaxation velocity (IVRTVel) showed poor agreement (ICC = 0.49 and 0.20). Accounting for the clustered data structure did not alter the findings: under an animal‐clustered bootstrap and after per‐animal aggregation, FHR agreement remained excellent (per‐animal ICC(A,1) 0.91; animal‐clustered 95% CI 0.88–0.96), and the agreement for all velocity and time‐interval metrics remained in the poor‐to‐moderate range.

**TABLE 3:** Agreement between manual spectral pulsed‐wave tissue Doppler imaging (M) and the automated color tissue Doppler imaging algorithm (A).

Metric	ICC(A,1) (95% CI)	ICC(C,1) (95% CI)	Bias (95% CI)	95% LoA	*N*	Prop. slope (95% CI)	*p*‐value	q (FDR)
*Duration*								
IVCT	0.50 (0.29–0.65)	0.63 (0.43–0.75)	6.72 (5.16, 8.28)	−8.49 to 21.93	95	−0.00 (−0.20, +0.19)	0.998	0.998
SmDur	0.63 (0.54–0.70)	0.64 (0.55–0.72)	−5.00 (−8.91, −1.09)	−43.11 to 33.10	95	−0.08 (−0.27, +0.11)	0.419	0.665
IVRT	0.45 (0.28–0.60)	0.57 (0.38–0.72)	14.85 (11.01, 18.68)	−22.34 to 52.03	94	**+0.33 (+0.12, +0.54)**	**0.002**	**0.012**
EmDur	0.53 (0.38–0.66)	0.71 (0.57–0.81)	−26.82 (−32.75, −20.90)	−74.20 to 20.55	64	**−0.37 (−0.56, −0.19)**	**<0.001**	**0.002**
AmDur	0.30 (0.16–0.42)	0.42 (0.22–0.61)	−10.19 (−12.99, −7.40)	−34.43 to 14.04	75	+0.16 (−0.13, +0.46)	0.281	0.563
FTDur	0.35 (0.18–0.49)	0.50 (0.24–0.71)	−35.03 (−44.05, −26.01)	−108.86 to 38.80	67	**−0.36 (−0.63, −0.09)**	**0.010**	**0.041**
*Velocity*								
SmVel	0.58 (0.39–0.70)	0.76 (0.61–0.84)	−2.40 (−2.83, −1.98)	−6.51 to 1.70	95	+0.16 (+0.01, +0.31)	0.034	0.101
EmVel	0.51 (0.36–0.63)	0.72 (0.57–0.82)	−2.17 (−2.59, −1.74)	−5.64 to 1.31	67	−0.06 (−0.26, +0.14)	0.576	0.691
AmVel	0.49 (0.31–0.61)	0.72 (0.52–0.83)	−3.42 (−4.06, −2.77)	−8.49 to 1.66	62	−0.14 (−0.34, +0.07)	0.190	0.457
IVCTVel	0.55 (0.34–0.68)	0.75 (0.58–0.84)	−2.79 (−3.25, −2.34)	−7.22 to 1.64	95	−0.06 (−0.21, +0.10)	0.453	0.665
IVRTVel	0.20 (0.05–0.33)	0.56 (0.22–0.71)	−2.17 (−2.40, −1.94)	−4.29 to −0.04	88	+0.02 (−0.21, +0.25)	0.888	0.968
*Fetal heart rate*								
FHR	0.94 (0.90–0.96)	0.94 (0.90–0.96)	−1.28 (−3.88, 1.33)	−26.63 to 24.08	95	−0.03 (−0.10, +0.05)	0.499	0.665

*Note*: Reported are intraclass correlation coefficients (ICC) with 95% confidence intervals (CIs): ICC(A,1) for absolute agreement and ICC(C,1) for consistency. Bland–Altman statistics include the mean bias (A − M) with its 95% CI and the limits of agreement (LoA, bias ±1.96 SD). Proportional bias is the slope of the Bland–Altman difference regressed on the mean, reported with its 95% CI and *p*‐value (test of slope ≠ 0); q is the Benjamini–Hochberg false discovery rate (FDR)‐adjusted value across all metrics, and bold indicates significant proportional bias (*q* < 0.05). Velocities are in cm/s, durations and intervals in ms, and fetal heart rate in bpm. *N* = number of paired observations.

### 3.2 Bias and limits of agreement (Bland–Altman analysis)

**FIGURE 5:**
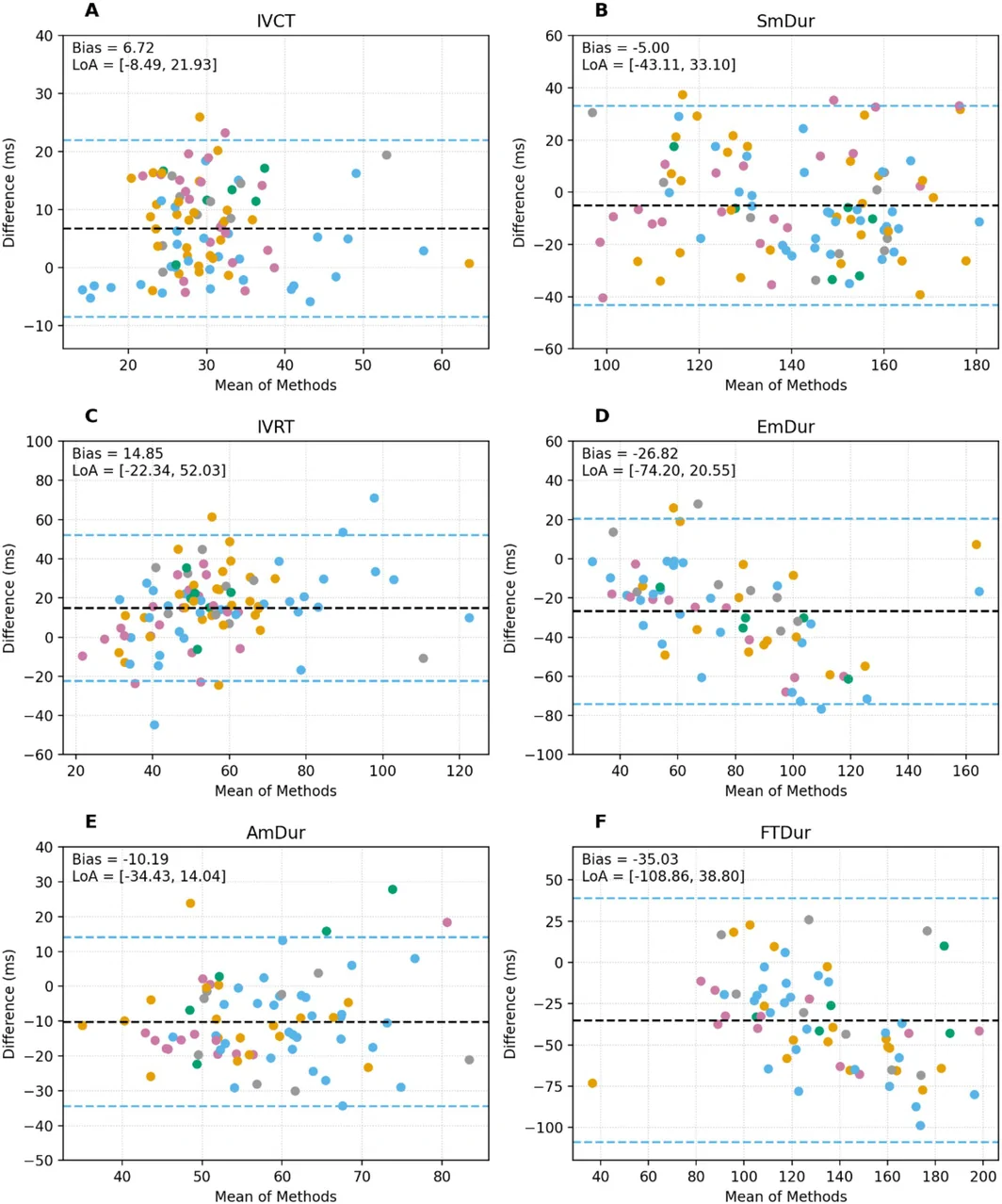
Bland–Altman plots for cardiac cycle time intervals comparing the automated analysis (A) with the manual observer (M). Each panel shows the difference A − M on the *y*‐axis against the mean (A + M)/2 on the *x*‐axis. The black dashed line marks the mean bias. The light‐blue dashed lines indicate the limits of agreement, calculated as bias ±1.96 SD. Points are color‐coded by experimental group and condition: Gray represents the right ventricle (RV) at baseline; orange, the RV during hypoxemia; sky blue, the RV in fetuses with aortic constriction (AoC); green, the left ventricle (LV) at baseline; and magenta, the LV during hypoxemia. Panels display, left to right and top to bottom: (A) IVCT, (B) SmDur, (C) IVRT, (D) EmDur, (E) AmDur, and (F) FTDur. Narrower limits of agreement (LoA) indicate better absolute agreement, whereas wider limits indicate greater variability between methods.

**FIGURE 6:**
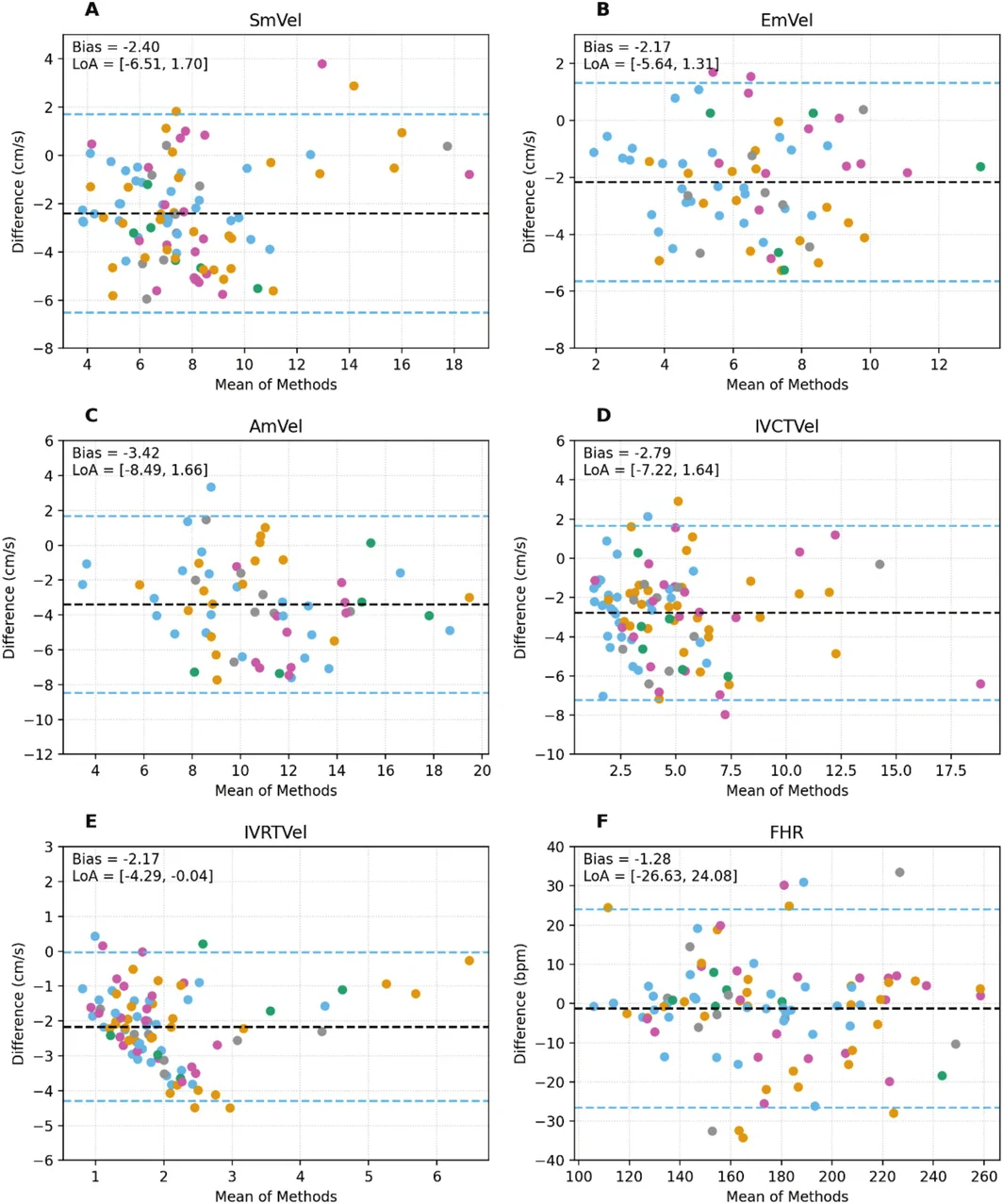
Bland–Altman plots for myocardial velocities and fetal heart rate comparing the automated analysis (A) with the manual observer (M). Each panel shows the difference A − M on the *y*‐axis versus the mean (A + M)/2 on the *x*‐axis. The black dashed line marks the mean bias, and the light‐blue dashed lines indicate the limits of agreement (bias ±1.96 SD). Points are color‐coded by experimental group and condition: Gray represents the right ventricle (RV) at baseline; orange, the RV during hypoxemia; sky blue, the RV in fetuses with aortic constriction (AoC); green, the left ventricle (LV) at baseline; and magenta, the LV during hypoxemia. Panels: (A) SmVel, (B) EmVel, (C) AmVel, (D) IVCTVel, (E) IVRTVel (all in cm/s), and (F) FHR (bpm). Narrower limits of agreement (LoA) indicate better absolute agreement; wider limits indicate greater variability between methods.

Bland–Altman analysis revealed systematic biases (Figures 5 and 6). For time intervals, the algorithm consistently overestimated isovolumic phases: IVCT showed a positive bias of +6.7 ms (95% LoA −8.5 to +21.9 ms) and IVRT a positive bias of +14.9 ms (95% LoA −22.3 to +52.0 ms). Conversely, diastolic filling durations were underestimated, with negative biases for EmDur (−26.8 ms), AmDur (−10.2 ms), and FTDur (−35.0 ms). Tissue velocities consistently showed a negative bias, indicating the algorithm measured lower peak velocities than the manual method. Biases ranged from −2.17 cm/s (EmVel and IVRTVel) to −3.42 cm/s (AmVel). FHR showed a small negative bias (−1.28 bpm) with limits of agreement, approximately −27 to +24 bpm.

### 3.3 Regression (Theil–Sen)

Robust regression analysis indicated varying proportional agreement. FHR showed a slope close to unity (0.94). For velocities, slopes generally ranged between 0.55 and 0.64, except for IVRTVel (0.13), reflecting the systematic underestimation of higher velocities. Time intervals showed dispersed slopes, ranging from 0.44 (FTDur) to 0.74 (IVRT), suggesting that the algorithm compresses the dynamic range relative to manual measurements for several metrics.

Proportional bias was assessed separately using Bland–Altman regression, defined as the slope of the between‐method difference regressed on the between‐method mean. After FDR correction, significant proportional bias was found for three time‐interval metrics: IVRT (+0.33, *q* = 0.012), EmDur (−0.37, *q* = 0.002), and FTDur (−0.36, *q* = 0.041). The negative slopes for EmDur and FTDur indicate greater underestimation at longer durations. SmVel showed a nominally significant unadjusted slope (*p* = 0.034), but this did not remain significant after FDR correction (*q* = 0.101). No other velocity metric and no FHR metric showed significant proportional bias.

### 3.4 Concordance (Lin's CCC)

Lin's CCC showed a similar pattern to the ICC findings. FHR demonstrated excellent concordance (CCC = 0.936). Among the other metrics, SmDur showed the highest concordance (CCC = 0.625), followed by SmVel (0.578) and IVCTVel (0.546). The remaining metrics showed lower concordance (CCC < 0.530), with IVRTVel showing the lowest agreement (CCC = 0.200).

### 3.5 Phase effects

At baseline, the modality offset was already present and significant. In both RV (Phase 0) and LV (Phase 3) the algorithm overestimated isovolumic intervals (IVCT, IVRT) and underestimated tissue velocities significantly for all RV velocities, and for most LV velocities (SmVel, IVCTVel, IVRTVel) in baseline (*q* < 0.05) whereas FHR showed no baseline bias (*q* > 0.78). None of the pre‐specified baseline‐versus‐hypoxemia contrasts reached significance (all *q* > 0.05), confirming that the offset was stable across different fetal oxygenation states.

## 4 DISCUSSION

In this study comparing automated cTDI with manual spectral pwTDI in a near‐term fetal sheep model under both physiological and pathological conditions, we found that FHR showed excellent agreement and near‐interchangeability between methods. In contrast, significant systematic offsets limited the absolute agreement for cardiac time intervals and myocardial velocities. These discrepancies are consistent with the fundamental physical and signal‐processing differences between the two modalities. While the automated algorithm preserved the relative ranking of individuals, its use for absolute measurements requires method‐specific baselines or calibration, particularly during physiological stress, such as hypoxemia.

The systematic offsets observed in this study are well explained by the different measurement principles and estimators used by pwTDI and cTDI. Spectral pwTDI samples a small tissue volume and uses fast Fourier transform–based spectral analysis to estimate a velocity spectrum.^30^ Clinical measurements typically rely on the spectral envelope or peak, which emphasizes the fastest‐moving myocardial components.

In contrast, cTDI derives a *mean* velocity per pixel using autocorrelation, followed by regional averaging. This process, combined with clutter filtering and spatial smoothing, systematically reduces measured velocity magnitudes compared with spectral peak values. Accordingly, our automated cTDI algorithm produced consistently lower myocardial velocities than manual pwTDI, consistent with previous findings in adults^30^, ^31^ and in human fetuses.^7^ Prior studies show that cTDI velocities are typically ~25% lower than pwTDI peak velocities.^30^, ^32^

Although several studies have evaluated myocardial peak velocities, no previous study has, to our knowledge, directly compared fetal cardiac cycle time intervals between spectral pwTDI and cTDI. In our data, timing metrics were affected in a similar manner to velocities. pwTDI typically identifies phase boundaries from velocity zero‐crossings, which serve as practical proxies for mechanical events.^33^, ^34^ The automated cTDI algorithm, however, identifies boundaries based on changes in velocity and its derivatives (acceleration and jerk), aligning with annular‐plane motion and valve transitions and operating without ECG input. Because these two approaches anchor intervals to different physiological features, their onsets do not necessarily coincide, resulting in the constant temporal offsets observed in isovolumic intervals. This also explains the proportional bias detected for several duration metrics, where waveform asymmetry produces magnitude‐dependent divergence between methods.

Differences in sampling also contribute to these discrepancies. pwTDI provides high temporal resolution at a single anatomical site, whereas cTDI reconstructs velocity fields at a lower frame rate and averages multiple pixels within the region of interest. Wall filtering and interpolation within the cTDI processing chain can broaden peaks and delay key landmarks. Taken together, these physical and processing differences fully explain the pattern observed in our results: lower absolute velocities and longer isovolumic times with automated cTDI, higher consistency than absolute agreement, and minimal bias for heart rate, which depends solely on temporal cycle counting.

Fetal heart rate presented a nuanced pattern of agreement. Although reliability was excellent (ICC ≈ 0.94) and the mean bias was negligible, the Bland–Altman analysis revealed wide limits of agreement (approximately −23 to +24 bpm). These findings highlight that, while the two methods are interchangeable on average at the population level, the absolute difference for an individual measurement can be substantial. Therefore, case‐by‐case interchangeability is limited, underscoring the importance of evaluating both ICC and absolute measurement error when assessing method agreement. The wide LoA are likely influenced not only by methodological differences but also by true physiological variability, as the two measurements were obtained sequentially rather than simultaneously.

The systematic offsets observed in this study have direct implications for derived indices, such as the Myocardial Performance Index (MPI), which incorporates IVCT, IVRT, and ejection time. Previous work has shown poor‐to‐moderate agreement in MPI across Doppler modalities in both adult and fetal populations.^35^, ^36^, ^37^ Our findings reinforce that automated, ECG‐independent phase extraction from cTDI must be rigorously validated before being used to derive clinically actionable MPI values for fetal assessment.

Previous studies have shown significant alterations in fetal cardiac function assessed with TDI in diabetic pregnancies,^38^ fetal growth restriction,^8^, ^39^ and hypoplastic left heart syndrome.^38^, ^40^ Across these conditions, TDI demonstrates high sensitivity for detecting even mild cardiac dysfunction and provides stronger predictive value for later cardiovascular complications than conventional ultrasound methods. However, these studies relied on spectral pwTDI, which is time‐consuming and typically limited to measurements obtained from only a few cardiac cycles. Automated cTDI, by contrast, offers a faster, more scalable approach to fetal cardiac evaluation.

Despite the wide limits of agreement for individual FHR measurements, the high reliability and negligible average bias indicate strong potential for automated cTDI as a tool for continuous, automated monitoring of FHR. As the resolution of cTDI is superior to that of conventional cardiotocography, the automated cTDI could be used as a complementary tool for higher quality monitoring of FHR and variability. The feasibility of extracting FHR variability from cTDI, even in early gestation, has been demonstrated previously,^41^ and warrants further exploration.

Conventional cardiac ultrasound parameters describing systolic and diastolic function—such as ejection fraction, ejection time, stroke volume, cardiac output, and fractional shortening—are known to be influenced by changes in loading conditions. Although TDI has been shown to be less load‐dependent,^3^, ^42^, ^43^ it remains essential to validate the applicability of automated cTDI measurements under both normal and pathological conditions.

A major strength of this study is the use of a fetal sheep model, which enabled evaluation of agreement under both physiological baseline conditions and perturbed states. Controlled experiments provided a robust test of the automated method across a spectrum of cardiac conditions which are not feasible to perform in human fetuses. However, due to species differences caution is needed while interpreting our results and findings may not be directly translated to a clinical setting.

Several limitations should be acknowledged. First, our findings are derived from a time‐dated fetal sheep model corresponding to near‐term human gestation (approximately 34–37 weeks), and generalization to earlier gestational ages or to human fetuses should be approached with caution—although the fetal cardiovascular system is mature in both species at this stage.^44^ There are some differences in cardiovascular physiology and anatomy between human and sheep fetuses. Compared with human, fetal sheep FHR is higher, but the combined cardiac output (CO) normalized to fetal weight^45^, ^46^, ^47^, ^48^ and ventricular pressures^49^, ^50^ are similar during the second half of pregnancy. The intra‐observer variabilities of Doppler ultrasonographic parameters of fetal sheep cardiovascular hemodynamics and tissue Doppler‐derived indices are comparable to those found in human fetuses during the second half of pregnancy.^51^, ^52^ Second, isovolumic velocities cannot be recorded by the automated algorithm used in this study. Finally, other potential sources of disagreement—such as small differences in sample placement or beat‐to‐beat variability—cannot be fully excluded. We attempted to minimize these by sampling similar myocardial regions at consistent insonation angles. At higher FHRs, fusion of the E and A diastolic peaks may also impair their detection. Averaging of the cardiac cycles may have affected the results and especially the LoA for the different parameters, as the automated algorithm uses several cardiac cycles (8.2 ± 2.9 beats) and for the manual analysis an average of three cardiac cycles was calculated.

## 5 CONCLUSION

Automated cTDI in sheep fetuses provided reproducible measurements of myocardial velocities that align well with manual pwTDI in terms of relative ranking, but absolute values were systematically lower. Cardiac cycle duration metrics showed larger systematic offsets and consequently poorer absolute agreement, reflecting inherent differences in signal processing and phase‐boundary definitions between the two modalities. For FHR, automated cTDI demonstrates excellent reliability on average, although individual measurement differences can still be substantial.

Taken together, these characteristics support the use of automated cTDI for longitudinal tracking, trend analysis, and comparative assessments of fetal sheep cardiac function—particularly when interpretation is anchored to method‐specific baselines or calibration rather than absolute thresholds. Future work should focus on refining automated phase detection to improve absolute agreement and on evaluating the generalizability of these findings in human fetuses across gestation.

## AUTHOR CONTRIBUTIONS

Conceptualization: JJ and GA. Methodology: JR, GA. Experimental animal work and data collection: JL, HH, MH, AB, JR, and GA. Data processing: MGM, JL, and JJ. Software: JJ. Data analysis; writing—original draft: JL and JJ. Supervision: GA. All authors reviewed and edited the manuscript. A large language model (Copilot) was used for grammar checking while preparing this manuscript.

## FUNDING INFORMATION

No specific funding was obtained.

## CONFLICT OF INTEREST STATEMENT

The author(s) declare no conflict of interests.

## ETHICS STATEMENT

This study was approved by the National Animal Experiment Board of Finland with reference ESAVI/1007/04.10.07/2014 on date March 13, 2014, and ESAVI/7840/04.10.07/2017 on date September 7, 2017.

## Data Availability

The datasets generated during and/or analyzed during the current study are available from the corresponding author on reasonable request.

## References

[1] Acharya G , Räsänen J , Kiserud T , Huhta JC . The fetal cardiac function. Curr Cardiol Rev. 2006;2(1):41‐53. doi:10.2174/157339406775471830

[2] Comas M , Crispi F . Assessment of fetal cardiac function using tissue Doppler techniques. Fetal Diagn Ther. 2012;32(1–2):30‐38. doi:10.1159/000335028 22626950

[3] Harada K , Tsuda A , Orino T , Tanaka T , Takada G . Tissue Doppler imaging in the normal fetus. Int J Cardiol. 1999;71(3):227‐234. doi:10.1016/S0167-5273(99)00152-7 10636528

[4] Paladini D , Lamberti A , Teodoro A , Arienzo M , Tartaglione A , Martinelli P . Tissue Doppler imaging of the fetal heart. Ultrasound Obstet Gynecol. 2000;16(6):530‐535. doi:10.1046/j.1469-0705.2000.00251.x 11169346

[5] Peixoto AB , Bravo‐valenzuela NJM , Martins WP , et al. Reference ranges of filling time and systolic‐to‐diastolic time index of the left ventricle, right ventricle, and interventricular septum using both spectral and tissue Doppler of fetal heart between 20 and 36 + 6 weeks of gestation. Eur J Obstet Gynecol Reprod Biol. 2020;252:366‐372. doi:10.1016/j.ejogrb.2020.07.017 32682211

[6] Peixoto AB , Bravo‐Valenzuela NJM , Mattar R , et al. Reference values for left and right ventricular systolic‐to‐diastolic duration ratio (SDR) found using both spectral and tissue Doppler of fetal heart between 20 and 36+6 weeks of gestation. Int J Cardiovasc Imaging. 2021;37(9):2717‐2726. doi:10.1007/s10554-021-02239-7 33844115

[7] Saini AP , Ural S , Pauliks LB . Quantitation of fetal heart function with tissue Doppler velocity imaging—reference values for color tissue Doppler velocities and comparison with pulsed wave tissue Doppler velocities. Artif Organs. 2014;38(1):87‐91. doi:10.1111/aor.12187 24117622

[8] Comas M , Crispi F , Cruz‐Martinez R , Martinez JM , Figueras F , Gratacós E . Usefulness of myocardial tissue Doppler vs conventional echocardiography in the evaluation of cardiac dysfunction in early‐onset intrauterine growth restriction. Am J Obstet Gynecol. 2010;203(1):45.e1‐45.e7. doi:10.1016/j.ajog.2010.02.044 20451892

[9] Larsen LU , Sloth E , Petersen OB , Pedersen TF , Sorensen K , Uldbjerg N . Systolic myocardial velocity alterations in the growth‐restricted fetus with cerebroplacental redistribution. Ultrasound Obstet Gynecol. 2009;34(1):62‐67. doi:10.1002/uog.6375 19489024

[10] Larsen LU , Petersen OB , Sloth E , Uldbjerg N . Color Doppler myocardial imaging demonstrates reduced diastolic tissue velocity in growth retarded fetuses with flow redistribution. Eur J Obstet Gynecol Reprod Biol. 2011;155(2):140‐145. doi:10.1016/j.ejogrb.2010.12.020 21256662

[11] Vyas HV , Eidem BW , Cetta F , et al. Myocardial tissue Doppler velocities in fetuses with hypoplastic left heart syndrome. Ann Pediatr Cardiol. 2011;4(2):129‐134. doi:10.4103/0974-2069.84650 21976871 PMC3180969

[12] Wohlmuth C , Wertaschnigg D , Wieser I , Arzt W , Tulzer G . Tissue Doppler imaging in fetuses with aortic stenosis and evolving hypoplastic left heart syndrome before and after fetal aortic valvuloplasty. Ultrasound Obstet Gynecol. 2016;47(5):608‐615. doi:10.1002/uog.14885 25914144

[13] Balli S , Pac FA , Ece İ , Oflaz MB , Kibar AE , Kandemir Ö . Assessment of cardiac functions in fetuses of gestational diabetic mothers. Pediatr Cardiol. 2014;35(1):30‐37. doi:10.1007/s00246-013-0734-0 23780554

[14] Hatém MAB , Zielinsky P , Hatém DM , et al. Assessment of diastolic ventricular function in fetuses of diabetic mothers using tissue Doppler. Cardiol Young. 2008;18(3):297‐302. doi:10.1017/S1047951108002138 18405423

[15] Herling L , Johnson J , Ferm‐Widlund K , et al. Fetal cardiac function at intrauterine transfusion assessed by automated analysis of color tissue Doppler recordings. Cardiovasc Ultrasound. 2020;18(1):34. doi:10.1186/s12947-020-00214-1 32792000 PMC7427079

[16] Acharya G , Huhta JC , Haapsamo M , How OJ , Erkinaro T , Räsänen J . Effect of angiotensin II on the left ventricular function in a near‐term fetal sheep with metabolic acidemia. J Pregnancy. 2011;2011:634240. doi:10.1155/2011/634240 22132338 PMC3206332

[17] Acharya G , Räsänen J , Mäkikallio K , et al. Metabolic acidosis decreases fetal myocardial isovolumic velocities in a chronic sheep model of increased placental vascular resistance. Am J Physiol Heart Circ Physiol. 2008;294(1):H498‐H504. doi:10.1152/ajpheart.00492.2007 18024549

[18] Hashima JN , Rogers V , Langley SM , et al. Fetal ventricular interactions and wall mechanics during ductus arteriosus occlusion in a sheep model. Ultrasound Med Biol. 2015;41(4):1020‐1028. doi:10.1016/j.ultrasmedbio.2014.11.002 25701524 PMC4407698

[19] Huhta H , Junno J , Haapsamo M , et al. Fetal sheep central haemodynamics and cardiac function during occlusion of the ascending aorta. Exp Physiol. 2018;103(1):58‐67. doi:10.1113/EP086500 29094424

[20] Lantto J , Erkinaro T , Haapsamo M , et al. Foramen ovale blood flow and cardiac function after main pulmonary artery occlusion in fetal sheep. Exp Physiol. 2019;104(2):189‐198. doi:10.1113/EP087423 30578690

[21] McDicken WN , Sutherland GR , Moran CM , Gordon LN . Colour doppler velocity imaging of the myocardium. Ultrasound Med Biol. 1992;18(6–7):651‐654. doi:10.1016/0301-5629(92)90080-t 1413277

[22] Herling L , Johnson J , Ferm‐Widlund K , Lindgren P , Acharya G , Westgren M . Automated analysis of color tissue Doppler velocity recordings of the fetal myocardium using a new algorithm. Cardiovasc Ultrasound. 2015;13(1):39. doi:10.1186/s12947-015-0034-3 26310927 PMC4549943

[23] Herling L , Johnson J , Ferm‐Widlund K , et al. Automated analysis of fetal cardiac function using color tissue Doppler imaging. Ultrasound Obstet Gynecol. 2018;52(5):599‐608. doi:10.1002/uog.18812 28715153

[24] Herling L , Johnson J , Ferm‐Widlund K , et al. Automated analysis of fetal cardiac function using color tissue Doppler imaging in second half of normal pregnancy. Ultrasound Obstet Gynecol. 2019;53(3):348‐357. doi:10.1002/uog.19037 29484743

[25] Finnish Government . FINLEX®—Translations of Finnish Acts and Decrees: 564/2013 English [Internet]. Oikeusministeriö; 2013. Accessed Aug 8, 2026. https://finlex.fi/en/laki/kaannokset/2013/20130564

[26] Government Decree on the Protection of Animals Used for Scientific or Educational Purposes (No. 564 of 2013). FAOLEX [Internet]. Accessed Aug 8, 2026. https://www.fao.org/faolex/results/details/en/c/LEX‐FAOC212156

[27] Directive ‐ 2010/63 ‐ EN ‐ EUR‐Lex [Internet]. Accessed Aug 8, 2026. https://eur‐lex.europa.eu/eli/dir/2010/63/oj

[28] du Sert NP , Ahluwalia A , Alam S , et al. Reporting animal research: explanation and elaboration for the ARRIVE guidelines 2.0. PLoS Biol. 2020;18(7):e3000411. doi:10.1371/journal.pbio.3000411 32663221 PMC7360025

[29] Bland JM , Altman DG . Measuring agreement in method comparison studies. Stat Methods Med Res. 1999;8(2):135‐160. doi:10.1177/096228029900800204 10501650

[30] Manouras A , Shahgaldi K , Winter R , Nowak J , Brodin LÅ . Comparison between colour‐coded and spectral tissue Doppler measurements of systolic and diastolic myocardial velocities: effect of temporal filtering and offline gain setting. Eur J Echocardiogr. 2009;10(3):406‐413. doi:10.1093/ejechocard/jen298 18988658

[31] Hummel YM , Klip IJT , de Jong RM , Pieper PG , van Veldhuisen DJ , Voors AA . Diastolic function measurements and diagnostic consequences: a comparison of pulsed wave‐ and color‐coded tissue Doppler imaging. Clin Res Cardiol. 2010;99(7):453‐458. doi:10.1007/s00392-010-0141-y 20221616 PMC2898098

[32] Abraham TP , Dimaano VL , Liang HY . Role of tissue Doppler and strain echocardiography in current clinical practice. Circulation. 2007;116(22):2597‐2609. doi:10.1161/CIRCULATIONAHA.106.647172 18040039

[33] Aase SA , Stoylen A , Ingul CB , Frigstad S , Torp H . Automatic timing of aortic valve closure in apical tissue Doppler images. Ultrasound Med Biol. 2006;32(1):19‐27. doi:10.1016/j.ultrasmedbio.2005.09.004 16364793

[34] Nestaas E , Schubert U , de Boode WP , El‐Khuffash A . Tissue Doppler velocity imaging and event timings in neonates: a guide to image acquisition, measurement, interpretation, and reference values. Pediatr Res. 2018;84(1):18‐29. doi:10.1038/s41390-018-0079-8 30072806 PMC6257218

[35] Acharya G , Pavlovic M , Ewing L , Nollmann D , Leshko J , Huhta JC . Comparison between pulsed‐wave Doppler‐ and tissue Doppler‐derived Tei indices in fetuses with and without congenital heart disease. Ultrasound Obstet Gynecol. 2008;31(4):406‐411. doi:10.1002/uog.5292 18340627

[36] Duzenli MA , Ozdemir K , Aygul N , Soylu A , Aygul MU , Gök H . Comparison of myocardial performance index obtained either by conventional echocardiography or tissue Doppler echocardiography in healthy subjects and patients with heart failure. Heart Vessel. 2009;24(1):8‐15. doi:10.1007/s00380-008-1069-2 19165562

[37] Rojo EC , Rodrigo JL , Pérez de Isla L , et al. Disagreement between tissue Doppler imaging and conventional pulsed wave doppler in the measurement of myocardial performance index. Eur J Echocardiogr. 2006;7(5):356‐364. doi:10.1016/j.euje.2005.08.004 16198634

[38] Patey O , Carvalho JS , Thilaganathan B . Perinatal changes in fetal cardiac geometry and function in diabetic pregnancy at term. Ultrasound Obstet Gynecol. 2019;54(5):634‐642. doi:10.1002/uog.20187 30520203

[39] Crispi F , Bijnens B , Sepulveda‐Swatson E , et al. Postsystolic shortening by myocardial deformation imaging as a sign of cardiac adaptation to pressure overload in fetal growth restriction. Circ Cardiovasc Imaging. 2014;7(5):781‐787. doi:10.1161/CIRCIMAGING.113.001490 24928572

[40] Natarajan S , Szwast A , Tian Z , McCann M , Soffer D , Rychik J . Right ventricular mechanics in the fetus with hypoplastic left heart syndrome. J Am Soc Echocardiogr. 2013;26(5):515‐520. doi:10.1016/j.echo.2013.02.001 23473605

[41] Zwanenburg F , Jongbloed MRM , van Geloven N , Ten Harkel ADJ , van Lith JMM , Haak MC . Assessment of human fetal cardiac autonomic nervous system development using color tissue Doppler imaging. Echocardiography. 2021;38(6):974‐981. doi:10.1111/echo.15094 34018638 PMC8252470

[42] Vogel M , Schmidt MR , Kristiansen SB , et al. Validation of myocardial acceleration during isovolumic contraction as a novel noninvasive index of right ventricular contractility. Circulation. 2002;105(14):1693‐1699. doi:10.1161/01.CIR.0000013773.67850.BA 11940549

[43] Vogel M , Cheung MMH , Li J , et al. Noninvasive assessment of left ventricular force‐frequency relationships using tissue Doppler–derived isovolumic acceleration. Circulation. 2003;107(12):1647‐1652. doi:10.1161/01.CIR.0000058171.62847.90 12668500

[44] Morrison JL , Berry MJ , Botting KJ , et al. Improving pregnancy outcomes in humans through studies in sheep. Am J Physiol‐Regul Integr Comp Physiol. 2018;315(6):R1123‐R1153. doi:10.1152/ajpregu.00391.2017 30325659

[45] Mielke G , Benda N . Cardiac output and central distribution of blood flow in the human fetus. Circulation. 2001;103(12):1662‐1668. doi:10.1161/01.CIR.103.12.1662 11273994

[46] Kiserud T . Fetal cardiac output, distribution to the placenta and impact of placental compromise. Ultrasound Obstet Gynecol. 2006;28:126‐136. doi:10.1002/uog.2832 16826560

[47] Anderson DF , Bissonnette JM , Faber JJ , Thornburg KL . Central shunt flows and pressures in the mature fetal lamb. Am J Phys. 1981;241(1):H60‐H66. doi:10.1152/ajpheart.1981.241.1.H60 7246789

[48] Rudolph AM . Distribution and regulation of blood flow in the fetal and neonatal lamb. Circ Res. 1985;57(6):811‐821. doi:10.1161/01.res.57.6.811 3905044

[49] Reller MD , Morton MJ , Reid DL , Thornburg KL . Fetal lamb ventricles respond differently to filling and arterial pressures and to in utero ventilation. Pediatr Res. 1987;22(6):621‐626. doi:10.1203/00006450-198712000-00001 3431944

[50] Johnson P , Maxwell DJ , Tynan MJ , Allan LD . Intracardiac pressures in the human fetus. Heart. 2000;84(1):59‐63. doi:10.1136/heart.84.1.59 10862590 PMC1729389

[51] Rasanen J , Wood DC , Debbs RH , Cohen J , Weiner S , Huhta JC . Reactivity of the human fetal pulmonary circulation to maternal hyperoxygenation increases during the second half of pregnancy: a randomized study. Circulation. 1998;97(3):257‐262. doi:10.1161/01.cir.97.3.257 9462527

[52] Bernard LS , Hashima JN , Hohimer AR , et al. Myocardial performance and its acute response to angiotensin II infusion in fetal sheep adapted to chronic anemia. Reprod Sci. 2012;19(2):173‐180. doi:10.1177/1933719111415545 22051849 PMC3343131

